# Health‐related quality of life in patients with chronic orofacial pain compared with other chronic pain patients

**DOI:** 10.1002/cre2.560

**Published:** 2022-03-28

**Authors:** Johanna Tanner, Tuija Teerijoki‐Oksa, Hannu Kautiainen, Pekka Vartiainen, Eija Kalso, Heli Forssell

**Affiliations:** ^1^ Department of Oral and Maxillofacial Diseases Turku University Hospital Turku Finland; ^2^ Department of Prosthetic Dentistry and Stomatognathic Physiology University of Turku Turku Finland; ^3^ Primary Health Care Unit Kuopio University Hospital Kuopio Finland; ^4^ Folkhälsan Research Center Helsinki Finland; ^5^ Department of Anaesthesiology, Intensive Care, and Pain Medicine, Division of Pain Medicine Helsinki University Hospital and University of Helsinki Helsinki Finland; ^6^ Department of Anaesthesiology, Department of Pharmacology, Intensive Care and Pain Medicine, Helsinki University Hospital SleepWell Research Programme University of Helsinki Helsinki Finland; ^7^ Department of Oral and Maxillofacial Surgery, Institute of Dentistry University of Turku Turku Finland

**Keywords:** 15D instrument, chronic pain, health‐related quality of life, orofacial pain, pain comorbidities, psychosocial factors

## Abstract

**Background:**

Health‐related quality of life (HRQoL) of orofacial pain patients is lower than that of the general population and impaired in multiple dimensions. The aim of the present study was to investigate HRQoL of orofacial pain patients in comparison with patients suffering from other chronic pain disorders.

**Materials and Methods:**

One hundred and fifty‐one tertiary care facial pain patients (mean age, 50 years; standard deviation [SD], 15; 119 females), were compared with 312 other non‐cancer chronic pain patients (mean age, 46 years; SD, 13; 204 women), recruited from three multidisciplinary pain clinics in Finland. The groups were compared using the 15D, and pain‐related measures such as pain interference, pain acceptance, anxiety, depression, and sleep. Statistical comparisons between groups were done using *t* test, *χ* ^2^ test, or analysis of covariance. Multivariate linear regression analysis was used to study whether pain‐related aspects influencing HRQoL are similar between the patient groups.

**Results:**

The 15D score was significantly higher in facial pain patients (0.823; SD, 0.114) indicating better HRQoL in comparison with other chronic pain patients (0.732; SD, 0.107) (*p* < .001). The 15D profiles of studied populations resembled each other but orofacial pain patients showed significantly higher scores for most individual 15D dimensions. Dimensions regarding discomfort and symptoms and sleep were most affected in both groups. Orofacial pain patients showed less psychosocial disability and better acceptance of their pain. Pain acceptance was a weaker explanatory factor of HRQoL in orofacial pain patients.

**Conclusion:**

Compared to other non‐cancer chronic pain, chronic pain in the orofacial area causes less impairment in HRQoL. Orofacial pain patients showed less psychosocial disability and better pain acceptance.

## 1 INTRODUCTION

Chronic pain affecting the orofacial region is defined as pain lasting for three months or more (Benoliel et al., 2019). It can be caused by either neuropathic, musculoskeletal, or neurovascular conditions (De Leeuw & Klasser, 2013). Prevalence of chronic orofacial pain is estimated to be approximately 8%–15% and it has notable socioeconomical impacts (Sessle, 2014). The most common causes of chronic orofacial pain are temporomandibular disorders (TMD). Burning mouth syndrome, neuropathic facial pain, persistent dentoalveolar pain disorder, and persistent idiopathic facial pain are also identified as common causes of chronic orofacial pain (Macfarlane et al., 2001).

Pain experience is complex, subjective, and difficult to describe and measure using solely conventional pain intensity scales (Turk & Okifuji, 2004). Chronic pain is related to physical health problems, psychological symptoms, and disability, and it thus impairs functioning in many aspects of everyday life (Breivik et al., 2006; Gatchel et al., 2007). Individual characteristics of a patient may influence how he or she reacts to and deals with a pain problem. Measuring health‐related quality of life (HRQoL) is a way to assess subjective experience of the adverse effects of a medical condition on the lives of those affected. HRQoL relates to physical, cognitive, and emotional functions and the ability to participate in meaningful activities within family, workplace, and community (Vetter, 2007). Measuring HRQoL offers health‐care professionals a wider understanding of patients’ preferences with regard to these various aspects of life and can therefore aid in the planning of the treatment for an individual patient (Borsook & Kalso, 2013; Turk et al., 2003). HRQoL is also concluded to be one of the core outcome indicators to be measured in the management of chronic pain (Kaiser et al., 2018; Turk et al., 2003).

Several different HRQoL instruments have been developed and they are categorized as being either generic or condition‐specific measures (Vetter, 2007). The number of studies addressing HRQoL and chronic orofacial pain is scarce and most of them have used condition‐specific HRQoL measures making it difficult to perform comparisons of chronic orofacial pain with other pain conditions.

The 15D instrument is a generic, self‐administered, standardized HRQoL instrument (www.15d‐instrument.net) that can be used both as a profile measure and as a single utility index measure, the 15D score (Sintonen, 2001). As a generic, preference‐based instrument it can be used to compare different patient populations. In a study on chronic pain the 15D has been shown to associate more strongly with pain severity than other commonly used HRQoL measures such as EQ‐5D (Vartiainen et al., 2017). Its use has also been validated in chronic orofacial pain (Forssell et al., 2020).

Studies using the 15D instrument in chronic non‐cancer pain patients have demonstrated a marked impairment of HRQoL of these patients, the 15D score being one of the lowest reported in any group of patients with chronic health problems (Dick et al., 2011; Vartiainen et al., 2016). The most affected dimensions of health in chronic pain are discomfort and symptoms, usual activities, sexual activity, vitality, and sleep (Vartiainen et al., 2016). Psychosocial factors appear to be more important to HRQoL than the intensity of chronic pain (Lamé et al., 2005; Orenius et al., 2013; Vartiainen et al., 2016).

The health‐related quality of life of orofacial pain patients has also been reported to be impaired (Forssell et al., 2020; Lopez‐Jornet et al., 2008; Smith et al., 2013). In our previous study using the 15D instrument, the mean 15D score of orofacial pain patients was found to be statistically and clinically significantly lower than that of an age‐ and gender standardized general population (Forssell et al., 2020). Whether the impact of chronic orofacial pain on HRQoL or pain‐related aspects influencing the HRQoL are similar to those of patients with chronic non‐cancer pain has not been studied.

The aim of the present study was to investigate HRQoL of chronic orofacial pain patients in comparison to patients suffering from other chronic pain disorders to gain insight into the similarities or differences between these patient groups. A further aim was to study whether there are some pain‐related aspects specific to orofacial pain which impair HRQoL. Our hypothesis was that HRQoL of chronic orofacial pain patients corresponds to that of other chronic non‐cancer pain patients.

## 2 METHODS

### 2.1 Subjects

This study is part of the multicenter study KROKIETA, chronic pain and lifestyle study in Finland. The material of the study consisted of 151 orofacial pain patients, who were compared with 312 patients suffering from other chronic non‐cancer pain conditions. The orofacial pain patients were recruited from facial pain clinics at three hospitals in Finland between 11/2013 and 11/2016. The patients with other types of chronic pain were recruited during the same time period from three multidisciplinary pain clinics in Finland. In both cases, consecutive patients with the age of 18–75 years, referred to pain management in tertiary care because of chronic pain, were invited to participate in the study. The patients were provided with written information about the study protocol. Patients who suffered from cancer‐related pain or were unable to answer the study questionnaire independently were not included. The study was approved by the Ethics Committee of the Helsinki University Hospital (decision no. 29/13/03/00/2012), and each hospital admitted permission for the study. An informed consent was obtained from all participants.

Among the recruited consecutive 164 orofacial pain patients, four patients refused to participate and nine patients did not return questionnaires or answered them incompletely. Finally, 151 orofacial pain patients were included in the study. For patients suffering from other pain conditions, formation of the study data set is described in previous studies reporting on the KROKIETA study (Miettinen et al., 2019, 2021; Vartiainen et al., 2017).

Results concerning HRQoL of the study patients in comparison to that of the general population have been published earlier (Forssell et al., 2020).

### 2.2 Measures

#### 2.2.1 Health‐related quality of life (HRQoL)

Health‐related quality of life (HRQoL) was measured using the 15D instrument. It is a self‐administered, standardized, generic, HRQoL instrument with 15 dimensions. It can be used both as a profile and a single index utility measure (Sintonen, 2001) (http://www.15d‐instrument.net/). It consists of 15 dimensions: mobility, vision, hearing, breathing, sleeping, eating, speech, excretion, usual activities, mental function, discomfort and symptoms, depression, distress, vitality, and sexual activity. For each dimension, the respondent chooses a statement about the severity of problems that best reflects his or her health state in that dimension. The valuation of the 15D is based on an application of the multi‐attribute utility theory, including a three‐stage valuation procedure performed in the general population. An overall single index score (the 15D score) and separate scores for individual dimensions are calculated based on mean dimension values. The maximum overall 15D score as well as the scores in individual dimensions are 1 (indicating no problems) and the minimum score is 0 (equal to being dead).

#### 2.2.2 Pain‐related measures

##### 2.2.2.1 Brief Pain Inventory (BPI)

BPI is a questionnaire designed to measure both pain intensity and the amount of interference pain has on functioning (Cleeland & Ryan, 1994; Tan et al., 2004). Pain intensity is measured with four items (worst, least, on average, and currently). Interference is measured with seven items, comprising general activity, mood, walking, work, relations with others, sleep, and enjoyment of life. Answers are given on a numerical rating scale (NRS) of 0–10, with the highest number indicating the worst imaginable pain for intensity items and complete interference for interference items.

##### 2.2.2.2 Mood

Depressive symptoms were measured using the Beck Depression Inventory‐II (BDI‐II) (Beck et al., 1961, 1996), which is one of the most commonly used measures to investigate depressive symptoms. It includes 21 items, which are scored on a scale from 0 to 3, with higher scores indicating more severe symptoms.

##### 2.2.2.3 Pain‐related anxiety

Pain‐related anxiety was assessed using the Pain Anxiety Symptoms Scale (PASS‐20) (McCracken, 2013), which is a 20‐item questionnaire addressing four anxiety subscales: cognitive anxiety, escape/avoidance behavior, fear of pain, and psychological anxiety symptoms. Subjects answer the items on a 6‐point scale ranging from “never” to “always.”

##### 2.2.2.4 Sleep

Sleep and sleeping disturbances were assessed with the Basic Nordic Sleep Questionnaire (BNSQ‐FIN2008) (Partinen & Gislason, 1995). Five multiple‐choice questions concerning difficulty to fall asleep, waking up during the night, use of sleep medication, and tiredness in the evening and in the morning, were chosen to be analyzed in this study. The individual questions were scored on scale from 0 to 4, with higher scores indicating increasing severity. A sum score of the five questions was used as a single index value.

##### 2.2.2.5 Pain acceptance

Chronic Pain Acceptance Questionnaire (CPAQ) (McCracken et al., 2004; Vowles et al., 2008) measures acceptance of chronic pain with two dimensions, namely activities and engagement ‐dimension and pain willingness ‐dimension. It consists of 20 items on a 7‐point scale ranging from “never true” to “always true.” Items on the pain willingness scale are reverse‐scored. Thus, increasing scores on both scales indicate increasing acceptance.

##### 2.2.2.6 Sociodemographic status

The sociodemographic status of subjects and information on health habits were assessed using questions based on FINRISK‐survey, a Finnish national health survey (Borodulin et al., 2015). Information on education years, marital status, employment, household income, smoking status, use of alcohol, and leisure time activities were extracted for the present study. Information on working status was allocated into three classes: still actively working, retired or unemployed. Information on marital status was allocated into two classes: cohabiting with someone or living alone. Smoking habits were classified into current smoking and nonsmoking. Use of alcohol was assessed using the Alcohol Use Disorders Identification Test (AUDIT), a 10‐question test with a 0–4 scale where larger numbers indicate more severe alcohol abuse behaviors. Leisure time physical activity was inquired as follows: “How often do you practice leisure time exercise at least 20 min so that you feel slightly breathless and sweat?” The information was allocated into three classes: less than once a week (low activity), one to three times a week (moderate activity), and more than three times a week (high activity).

### 2.3 Study design

Subjects were asked to fill in the above‐mentioned questionnaires before or at their first visit to the facial pain or multidisciplinary pain clinics.

The HRQoL of patients suffering from chronic orofacial pain was compared, using the 15D score and individual profile scores, with that of the patients suffering from other chronic non‐cancer pain. To study possible differences between the patient populations in factors explaining the impairment of HRQoL, the relationship between individual pain‐related measures and 15D score was studied.

#### 2.3.1 Statistical analyses

Data are expressed as the mean and standard deviation (SD) and counts with percentages. Statistical comparisons between groups were done using *t* test, *χ* ^2^ test, or analysis of covariance. Multivariate linear regression analysis was used to identify the relationship between 15D score as continuous variables and the pain‐related measures with standardized regression coefficient *β*. Models included age, gender, and education years as covariates. The *β* value is a measure of how strongly the predictor variable influences the criterion variable. The *β* is measured in units of SD. Cohen's standard for *β* values above .10, .30, and .50 represent small, moderate, and large relationships, respectively (Cohen, 1988). In the case of violation of the assumptions (non‐normality), a bootstrap‐type or permutation‐type test were used. The normality of variables was evaluated graphically and using the Shapiro–Wilk test. All reported *p* values are two sided, and statistical significance (*α* level) was set at .05 for all tests. All analyses were performed using STATA software, version 16.1 (StataCorp LP, College Station, TX).

## 3 RESULTS

The two studied patient populations were similar with regard to age, education, and other socioeconomic factors. Facial pain patients were more often female than the other pain patients. Duration of pain in the patient populations was found to be very similar, the majority of patients in both groups had suffered from pain disorders for more than 2 years (Table 1).

**Table 1:** Sociodemographic and pain‐related psychosocial characteristics of the studied patient populations

	Muu (*N* = 312)	Kasvo (*N* = 151)	*p* Value
Number of female, *n* (%)	204 (65)	119 (79)	.003
Age, mean (SD)	46 (13)	50 (15)	.006
Education years, mean (SD)	13.3 (3.0)	13.8 (3.8)	.11
Working, *n* (%)	142 (46)	80 (53)	.13
Cohabiting, *n* (%)	193 (62)	104 (69)	.14
Household income^a^ 1000€, mean (SD)	30 (16)	29 (17)	.67
Current smoking, *n* (%)	121 (39)	23 (15)	<.001
AUDIT, mean (SD)	3.9 (4.3)	2.5 (2.7)	<.001
Leisure‐time physical activity (LTPA), *n* (%)			.077
Low	129 (41)	46 (30)	
Moderate	117 (38)	67 (44)	
High	66 (21)	38 (25)	
BNSQ sum, mean (SD)	11.6 (4.1)	9.0 (4.5)	<.001
BDI, mean (SD)	15.8 (9.7)	9.7 (8.3)	<.001
CPAQ, mean (SD)	52.6 (17.8)	64.7 (19.8)	<.001
PASS, mean (SD)	44.6 (18.9)	37.6 (18.8)	<.001
BPI/intensity, mean (SD)	5.9 (1.6)	4.9 (2.0)	<.001
BPI/interference, mean (SD)	6.4 (1.9)	4.2 (2.5)	<.001
Duration of pain, *n* (%)			.56
<1 year	42 (13)	23 (17)	
1–2	43 (14)	20 (13)	
>2	227 (73)	105 (70)	

Abbreviations: BDI, Beck Depression Inventory; BNSQ, Basic Nordic Sleep Questionnaire; BPI, Brief Pain Inventory; CPAQ, Chronic Pain Acceptance Questionnaire; PASS, Pain Anxiety Symptoms Scale.

^a^ Household income by the square root of household size; Euro 2017.

Lifestyle of the facial pain patients was generally more favorable compared with patients suffering from other chronic pain conditions. Facial pain patients smoked less, consumed less alcohol, and were somewhat more physically active at leisure time. The household income level did not differ between the groups. When compared with the other pain patients, facial pain patients had less problems related to sleep, they reported less depressive and pain‐related anxiety symptoms and showed better acceptance of pain. Pain intensity and interference scores were also lower in the group of patients with facial pain. Comparison of demographic and socioeconomic data, lifestyle factors, and pain‐related measures are presented in detail in Table 1.

**Figure 1:**
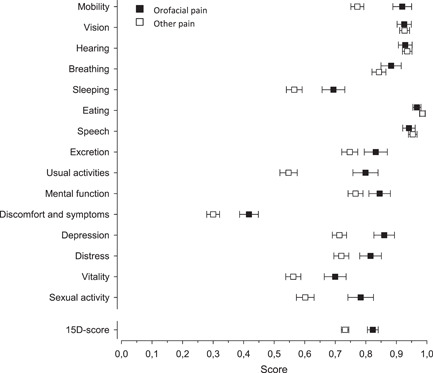
HRQoL of patients suffering from orofacial pain and other chronic pain condition measured with the 15D instrument. Means with 95% confidence intervals are shown. HRQoL, health‐related quality of life

The 15D score was significantly higher in facial pain patients (0.823) compared with the other chronic pain patients (0.732) (*p* < .001), indicating a better HRQoL for patients with orofacial pain in comparison with the other chronic pain patients (Table 2). For most individual 15D dimensions (mobility, breathing, sleeping, exertion, usual activities, mental function, discomfort and symptoms, depression, distress, vitality, and sex) orofacial pain patients showed significantly higher scores than patients with other pain conditions. For the dimensions vision, hearing and speech, the studied patient populations demonstrated similar profiles, whereas for the dimension eating, statistically significantly lower scores were measured for orofacial pain patients. The mean 15D profile of orofacial pain patients compared with that of chronic non‐cancer pain patients is presented in Figure 1.

**Table 2:** The mean (95% CI) values of the 15D score and the scores for individual dimensions of the 15D of patients with chronic orofacial pain (*N* = 151) and patients with other chronic non‐cancer pain (*N* = 312)

	Chronic orofacial pain, mean (SD)	Other chronic non‐cancer pain, mean (SD)	*p* Value^a^
Mobility	0.915 (0.152)	0.775 (0.208)	<.001
Vision	0.925 (0.165)	0.927 (0.132)	.94
Hearing	0.929 (0.138)	0.936 (0.142)	.65
Breathing	0.885 (0.185)	0.842 (0.217)	.054
Sleeping	0.693 (0.258)	0.566 (0.214)	<.001
Eating	0.967 (0.103)	0.985 (0.071)	.031
Speech	0.943 (0.131)	0.953 (0.123)	.32
Excretion	0.831 (0.210)	0.748 (0.243)	<.001
Usual activities	0.800 (0.244)	0.546 (0.256)	<.001
Mental function	0.850 (0.204)	0.764 (0.224)	<.001
Discomfort and symptoms	0.418 (0.222)	0.300 (0.171)	<.001
Depression	0.863 (0.178)	0.713 (0.220)	<.001
Distress	0.824 (0.208)	0.716 (0.227)	<.001
Vitality	0.703 (0.221)	0.561 (0.220)	<.001
Sexual activity	0.787 (0.263)	0.600 (0.259)	<.001
15D‐score	0.824 (0.114)	0.732 (0.107)	<.001

Abbreviation: CI, confidence interval.

^a^ Adjusted for age, gender, and education years.

Table 3 shows the results of the regression analysis. Each of the pain‐related measures had a statistically significant impact on the 15D score in both patient groups. There were no significant differences between the patient groups except for chronic pain acceptance (CPAQ) which showed a statistically significantly stronger correlation with 15D score of the other chronic non‐cancer pain patients than with the facial pain patients. For other pain‐related measures (PASS, BDI, BNSQ, and BPI) the correlations did not differ statistically significantly between the studied populations, although for depressive symptoms there was a trend toward stronger correlation with the 15D score of facial pain patients.

**Table 3:** Relationship between pain‐related measures and 15D score

	Other chronic non‐cancer pain, *β* (95% CI)^a^	Chronic orofacial pain, *β* (95% CI)^a^	*p* value**
CPAQ	.65 (0.56 to 0.75)	.49 (0.37 to 0.60)	.026
PASS	−.51 (−0.60 to −0.42)	−.51 (−0.64 to −0.38)	.98
BDI	−.62 (−0.70 to −0.54)	−.76 (−0.89 to −0.61)	.074
BNSQ	−.49 (−0.58 to −0.40)	−.61 (−0.73 to −0.49)	.13
BPI			
Intensity	−.40 (−0.51 to −0.29)	−.47 (−0.60 to −0.35)	.36
interference	−.66 (−0.76 to −0.55)	−.62 (−0.74 to −0.51)	.66

*Note: β* indicates regression coefficient between the studied variable and the 15D score.

Abbreviations: BDI, Beck Depression Inventory; BNSQ, Basic Nordic Sleep Questionnaire; BPI, Brief Pain Inventory; CPAQ, Chronic Pain Acceptance Questionnaire; PASS, Pain Anxiety Symptoms Scale.

^a^ Standardized *β*. Adjusted for age, gender education years.

** The *p* value shows the statistical significance of the difference between the *β* coefficients.

## 4 DISCUSSION

This cross‐sectional observational study investigated the health‐related quality of life (HRQoL) of chronic orofacial pain patients in comparison with patients suffering from other chronic non‐cancer pain conditions. In addition to HRQoL, pain‐related measures, such as pain intensity and interference, pain acceptance, pain‐related anxiety, depression, and sleep, were investigated. On the whole, orofacial pain patients were found to perceive their HRQoL better than other chronic non‐cancer pain patients. The studied patient groups demonstrated similar profiles of the 15D, but the scores of orofacial pain patients were statistically significantly higher regarding almost all dimensions of HRQoL. Only for the dimension regarding eating, did orofacial pain patients report significantly lower scores. When compared with the other pain patients, the facial pain patients reported lower pain intensity and interference, less depressive and pain‐related anxiety symptoms, and had less problems related to sleep, but showed better acceptance of pain. No significant differences were noted in the influence of these pain‐related aspects on quality of life except for pain acceptance that seemed to be a stronger explanatory factor of HRQoL in patients with other chronic pain problems compared to orofacial pain patients.

Based on the findings of the present study, chronic pain in the orofacial region seems to cause less burden as regards quality of life and psychosocial wellbeing than other chronic pain conditions. An explanation behind this difference may be that bodily pains are likely to have a more severe effect on for example, mobility and usual activities, compared with those caused by orofacial pain conditions. Health‐related quality of life of chronic pain patients is found to be more impaired in patients suffering from widespread pain compared with patients with only few pain locations (Lamé et al., 2005). Also, musculoskeletal pain in the craniomandibular region has previously been found to impair HRQoL to a smaller extent than a more widespread pain (Lobbezoo et al., 2004). In any case, according to the present and previous study (Forssell et al., 2020), orofacial pain affects a broad spectrum of health dimensions, emphasizing the importance of comprehensive assessment and care in chronic orofacial pain.

Using the 15D instrument to measure HRQoL enables comparison of different patient populations with regard to quality of life and its different dimensions. In the present study orofacial pain patients received a higher score for both the 15D score and for nearly all individual dimensions of the 15D instrument. In general, the 15D profiles of the two studied patient groups resembled each other, with both populations showing their lowest scores for discomfort and symptoms, which is in line with previous findings (Dick et al., 2011; Vartiainen et al., 2016, 2019). Both patient groups reported distinct impairments also for the dimensions regarding vitality and sleep. For patients with other pain conditions, the ability to perform usual daily activities, such as work assignments, studies, and housework, was also strongly negatively affected. The largest difference between the two chronic pain groups was in the “daily activities” dimension, followed by “sexual activity,” “mobility,” “depression,” and “sleep.” The dimensions regarding vision, hearing, eating, and speech were least affected for both populations. Interestingly, for the dimension regarding eating, and to a smaller extent also the dimensions speech and hearing, orofacial pain patients received lower scores than patients with other pain conditions. This can be attributed to pain location close to anatomical structures responsible for these functions. Pain located in the jaws and facial region is likely to cause disturbance in oral functions such as speech and eating. TMD pain is often located in the periauricular area and associated with otological comorbidities, such as tinnitus and may thus be reflected on the dimension regarding hearing (Skog et al., 2019).

The studied patient populations did not differ with regard to sociodemographic variables, except that facial pain patients were more often females. Instead, differences were seen in health habits and psychological symptoms. Orofacial pain patients lead a generally more active and healthy lifestyle. In addition to a more favorable health behavior, such as low consumption of tobacco and alcohol, orofacial pain patients were more physically active at leisure time. Healthier lifestyle is often linked to better socioeconomic position, though here the household income levels were identical in studied patient groups. Previous studies have associated higher pain interference to lower recreational activity (Karoly & Ruehlman, 2007) and increased nicotine and alcohol dependence (McDermott et al., 2018). In line, patients with orofacial pain had a healthier lifestyle and experienced lower pain intensity and their pain interfered less with their lives. This healthier and more active lifestyle of the orofacial pain patients might also account for the higher HRQoL scores, although causational relationship cannot be determined from this cross‐sectional study.

Chronic pain and psychological problems often occur simultaneously and overlap. In the present study orofacial pain patients showed lower psychosocial disability and better acceptance of their pain compared to other chronic pain patients. Acceptance of pain is generally viewed as willingness to experience continuing pain without the need to control it (McCracken, 1999) and is associated with lower levels of pain and better physical, social and emotional functioning (McCracken & Eccleston, 2003; Viane et al., 2003). Poor acceptance of pain has previously been associated with a lower quality of life and an unfavorable treatment outcome of chronic pain patients (Miettinen et al., 2019). Ojala et al. (2013) found a correlation of CPAQ to HRQoL, which associated with the activities and engagement‐dimension of CPAQ.

Results of the regression analysis indicated that all studied pain‐related measures had a strong impact on HRQoL. The two patient groups were similar regarding the impact of these factors except for pain acceptance, which was a stronger explanatory factor for the HRQoL outcome of patients with other chronic pain. These patients showed poorer acceptance of pain and their HRQoL was more impaired. This difference in *β* coefficients might indicate that pain acceptance is more important for the overall well‐being in other chronic pain than in orofacial pain, or that the relationship of pain acceptance and HRQoL is not linear, that is, the interdependency is stronger in more severe symptoms. Since facial pain patients had more favorable results in most of the measured aspects, their perceived better HRQoL is likely to be a net effect of several of these or other factors not covered in this study.

### 4.1 Methodological considerations

The groups of patients were similar with regard to pain duration. In both groups over 70% of patients had experienced pain for more than 2 years, indicating that both patient groups represented patients with long‐lasting chronic pain problems. A limitation of the study was that information on comorbid pain conditions or other medical comorbidities were not assessed. Many chronic medical conditions are associated with chronic pain and the number of comorbidities has been shown to correlate with HRQoL of chronic noncancer pain patients (Dick et al., 2011). Comorbidities related to other diseases may have accounted for some of the variation in the scores of the present study as well. Patients with orofacial pain reported pain in other areas of the body as well (Forssell et al., 2020), though their main complaint was pain in the facial area.

The 15D instrument was chosen to be used in this study because its validity in the study of chronic pain has been demonstrated in previous research (Vartiainen et al., 2016, 2017). Its usefulness in orofacial pain is supported by a study demonstrating convergent validity between 15D and pain interference in orofacial pain patients (Forssell et al., 2020).

A possible disadvantage of the present study is the use of a generic HRQoL instrument. It is known to be less sensitive than a condition‐specific instrument and may thus not distinguish all relevant features of the disease. However, using a generic instrument enabled us to make comparisons of HRQoL with a patient population suffering from other pain conditions.

## 5 CONCLUSIONS AND CLINICAL IMPLICATIONS

Chronic pain in the orofacial area causes less impairment in HRQoL than does other chronic non‐cancer pain. Furthermore, the patients have a lower pain burden in general; they suffer less from psychosocial disability, show better pain acceptance and engage a healthier lifestyle. There are no differences in the pain‐related factors influencing HRQoL in the two patient groups except for pain acceptance; pain acceptance explains the HRQoL more strongly in patients with other chronic pain problems compared to orofacial pain patients. Despite the findings of better HRQoL and psychosocial functioning in orofacial pain patients in comparison with other chronic pain patients, the results of this study are in line with earlier studies in showing that orofacial pain affects a broad spectrum of health dimensions. This emphasizes the importance of comprehensive assessment and care in chronic orofacial pain. The present results might, however, indicate that not all patients with chronic orofacial pain need as comprehensive and as multidimensional care as is often indicated for patients with other non‐cancer pain problems, but this needs corroboration.

## AUTHOR CONTRIBUTIONS

Johanna Tanner drafted and edited the manuscript and participated in the analysis of the data. Tuija Teerijoki‐Oksa collected the data and participated in manuscript editing. Hannu Kautiainen analyzed the data and participated in drafting and editing the manuscript. Pekka Vartiainen participated in manuscript editing. Eija Kalso and Heli Forssell conceptualized the study, defined the methodology, and participated in data analysis and manuscript editing.

## CONFLICTS OF INTEREST

The authors declare no conflicts of interest.

## Data Availability

The data that support the findings of this study are available from the corresponding author upon reasonable request.
